# A Comparative Study of Soy Protein Isolate-κ-Carrageenan Emulsion Gels and Bigels for the Encapsulation, Protection, and Delivery of Curcumin

**DOI:** 10.3390/gels11100782

**Published:** 2025-09-30

**Authors:** Emmanueline T Gray, Weining Huang, Zhongkai Zhou, Hao Cheng, Li Liang

**Affiliations:** 1 State Key Laboratory of Food Science and Resources, School of Food Science and Technology, Jiangnan University, Wuxi 214122, China; 6230112907@stu.jiangnan.edu.cn (E.T.G.); wnhuang@jiagnan.edu.cn (W.H.); liliang@jiangnan.edu.cn (L.L.); 2 School of Food Science and Technology, Shihezi University, Shihezi 832000, China; zkzhou@shzu.edu.cn

**Keywords:** bigels, emulsion gels, soy protein isolate, κ-carrageenan, curcumin, delivery

## Abstract

Protein-based emulsion gels and bigels serve as ideal delivery systems owing to their distinctive structural properties, high encapsulation efficiency, and adjustable digestive behavior. However, limited research has examined the differences between emulsion gels and bigels as polyphenol delivery systems. In this study, oil-in-water (O/W)-type emulsion gels formulated with soy protein isolate (SPI) and κ-carrageenan (κ-CG) were fabricated using a cold-set gelation method, and then the bigels were prepared through further oil gelation by the addition of glycerol monostearate (GMS). Both SPI-κ-CG emulsion gels and bigels were mainly stabilized by electrostatic and hydrophobic interactions, exhibiting high gel strength, varying from 940 g to 1304 g, and high water holding capacity (~84%). Both the SPI-κ-CG emulsion gels and bigels demonstrated high curcumin encapsulation efficiency, reaching 98~99%. Stability testing revealed that bigels prepared with 15% and 20% GMS exhibited the highest curcumin retention ratios, with a value of around 78% after storage for 21 days at 25 °C, suggesting that denser network structures more effectively prevent the degradation of the encapsulated compound. During the in vitro simulated gastric digestion, higher GMS content significantly delayed curcumin release by over 7%. Increasing GMS concentration from 0% to 20% elevated lipolysis by over 8% and concurrently improved the release of curcumin by more than 18% during the in vitro simulated intestinal digestion. This study provides comparative insights into polyphenol delivery performance between emulsion gels and bigels, offering valuable guidance for developing functional foods based on gel delivery systems.

## 1. Introduction

Curcumin, a natural polyphenol characterized by its bis-feruloylmethane structure, demonstrates multiple pharmacological activities including antioxidant, anti-inflammatory, anticancer, hepatoprotective, and anti-atherosclerotic effects [1]. Recent studies have demonstrated that the health benefits of curcumin result from its numerous active metabolites transformed by the host and microbial enzymes [2,3]. However, its poor aqueous solubility, sensitivity to environmental factors, and gastrointestinal tract conditions (e.g., oxygen, light, heat, enzymes) limit its application in functional foods [4]. Therefore, developing edible carriers is crucial to enhancing curcumin’s physicochemical stability and targeted release properties.

To date, food gels have gained considerable interest owing to their distinctive thermodynamic stability, tunable rheological properties, and controlled-release capabilities [5]. Among these gel systems, emulsion-filled gels consisting of emulsified oil droplets entrapped within a hydrogel have been developed for the encapsulation, protection, and delivery of both hydrophobic and hydrophilic bioactives because they combine the advantages of both emulsion and gel network characteristics [6,7,8,9,10]. Within emulsion gel matrices, protein-bound oil droplets functionally integrate bioactive encapsulation with structural modulation of the 3D network [11]. This configuration enhances protection efficacy for oil-phase bioactives through compartmentalization effects and achieves spatiotemporally programmed release during gastrointestinal transit [12,13].

Bigels possess a novel biphasic system integrating both hydrogel and oleogel phases, making them serve as novel carriers, 3D printing materials, and fat replacers [14]. The bigels with an oleogel-in-hydrogel microstructure can serve as a special form of emulsion-filled gels, exhibiting higher stability due to both gelled phases in the gel network [15]. The gelled oil phase in bigels not only protects encapsulated ingredients against environmental stressors (e.g., oxidation, humidity) but also critically governs microstructure and mechanical properties. By modulating the oleogel’s composition, such as crystalline network density or oleogelator concentration, the entire bigel system can be engineered to optimize loading capacity and achieve stimuli-responsive release kinetics for incorporated bioactives [14]. Lu and co-workers fabricated oleogel-in-hydrogel bigels for the co-delivery of epigallocatechin gallate and curcumin [16]. It was found that the increase in oleogelator concentration resulted in more compact gel networks, leading to a lower release rate of curcumin in gastric digestion but a higher release rate in the intestinal stage. In another study, bigels with konjac glucomannan-gelatin hydrogel phase and stearic acid oleogel phase were fabricated for the delivery of curcumin, and found that the increase in oleogel/hydrogel volume ratio could achieve a sustained free fatty acid release but decrease the bioaccessibility of curcumin [17]. Bigels have been also used for the encapsulation and delivery of other polyphenols, such as quercetin, catechin [18], and epigallocatechin gallate [19]. These bioactive-loaded bigel systems show great promise as functional ingredients for use as fat replacers, in food packaging applications, and in 3D printing foods [20]. Recently, Kaimal and co-workers compared the difference in the encapsulation of ascorbic acid within emulsion gels and bigels formulated with xanthan-guar gum and ethyl cellulose; they found that bigels and emulsion gels had the same bioaccessibility of ascorbic acid with a value of 87% [21]. While oleogel-in-hydrogel bigels, with their structured oil phase, are theoretically expected to provide enhanced protection and tunable release profiles for bioactives compared to emulsion-filled gels, direct comparative studies between these two systems remain scarce.

Proteins and polysaccharides are widely used in the formation of food gels. The combination of these two biomacromolecules can lead to distinctive structural and functional properties, owing to the interactions between proteins and polysaccharides [22]. Soy protein isolate (SPI), a commercially significant food protein, is widely recognized for its nutritional value, functional properties, and health benefits. κ-Carrageenan (κ-CG), a linear sulfated polysaccharide (15~40% ester sulfate), consists of alternating α-(1→3)-D-galactopyranose and β-(1→4)-3,6-anhydro-D-galactopyranose units. Our previous studies demonstrated that protein-κ-CG hydrogels sequentially crosslinked by KCl and glucono-δ-lactone (GDL) showed enhanced mechanical strength and decreased swelling ratio. In this study, SPI-κ-CG emulsion-filled gels were fabricated using the double crosslinking gelation method. Glycerol monostearate (GMS) with various concentrations was further used as an oleogelator to prepare SPI-κ-CG bigels. This study systematically investigated structural evolution mechanisms, microstructural architectures, textural profiles, and in vitro digestive behaviors to elucidate functional divergences between emulsion gels and bigels in encapsulating, protecting, and delivering curcumin. Our research establishes a theoretical framework for selecting optimal gel carriers to enhance hydrophobic polyphenol delivery in nutraceutical applications.

## 2. Results and Discussion

### 2.1. Size Distribution and ζ-Potential of Emulsions

**Figure 1:**
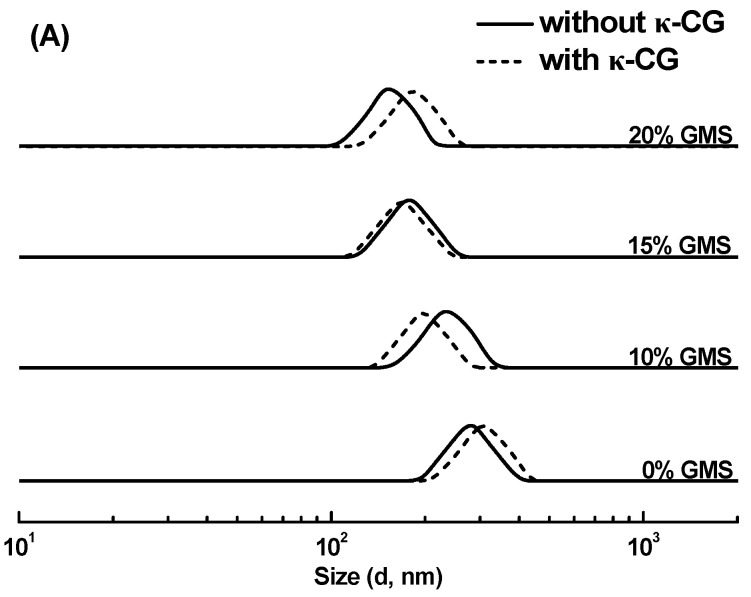

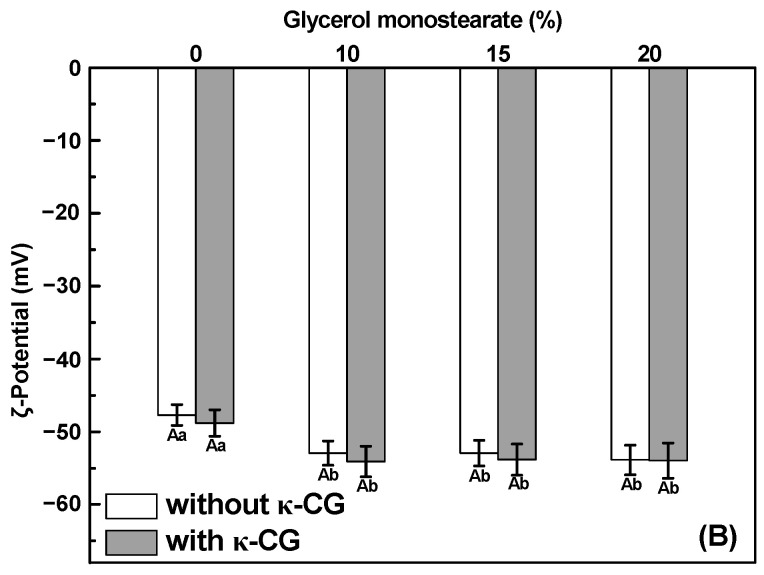
The size distribution (**A**) and ζ-potential (**B**) of soy protein isolate-stabilized emulsions with various glycerol monostearate (GMS) concentrations in the absence and presence of 0.25% κ-carrageenan (κ-CG). Different letters (uppercase letters for samples with or without κ-CG, lowercase letters for GMS concentration) indicate statistically significant differences (*p* < 0.05).

As shown in Figure 1A, all SPI-stabilized emulsions with various GMS concentrations in the absence and presence of κ-CG exhibited a unimodal size distribution. SPI-stabilized emulsions without GMS exhibited the largest droplet size with a peak of around 300 nm. The addition of GMS resulted in the gradual decrease in oil droplet size of emulsions, with a peak of around 150 nm at 20% GMS. These results suggested that the addition of GMS facilitates smaller oil droplet formation. This was attributed to the high surface activity of GMS with low molecular weight absorbing faster than SPI into the water–oil interface during the homogenization [23]. Additionally, unlike sunflower oil, which consists largely of triacylglycerides, the GMS molecule lacks esterified fatty acyl groups and is more hydrophilic. This property causes a substantial reduction in the interfacial tension of the oil phase, thereby decreasing the size of emulsified oil droplets [24,25]. The addition of 0.25% κ-CG had a slight impact on the size distribution of emulsions, which was observed in the pea protein-κ-CG sunflower oil emulsions and whey protein-gum Arabic sunflower oil emulsions due to the weak interaction between the protein and anion polysaccharide in the neutral environment [13,26].

SPI-stabilized emulsions exhibited a ζ-potential of approximately −48 mV (Figure 1B). Since the emulsion pH (~7.0) exceeded the isoelectric point of SPI (~4.5) [27], the emulsified oil droplets possessed negatively charged surfaces. Adding GMS slightly increased the ζ-potential of the droplets (e.g., −54 mV at 20% GMS), possibly due to altered protein adsorption at the oil–water interfaces [24]. However, incorporating κ-CG into the SPI-stabilized emulsion had no significant effect (*p* < 0.05) on ζ-potential. This suggests that the negatively charged κ-CG molecules remained primarily in the aqueous phase and did not adsorb onto the oil droplet surfaces.

### 2.2. Gel Formation and Microstructure

**Figure 2:**
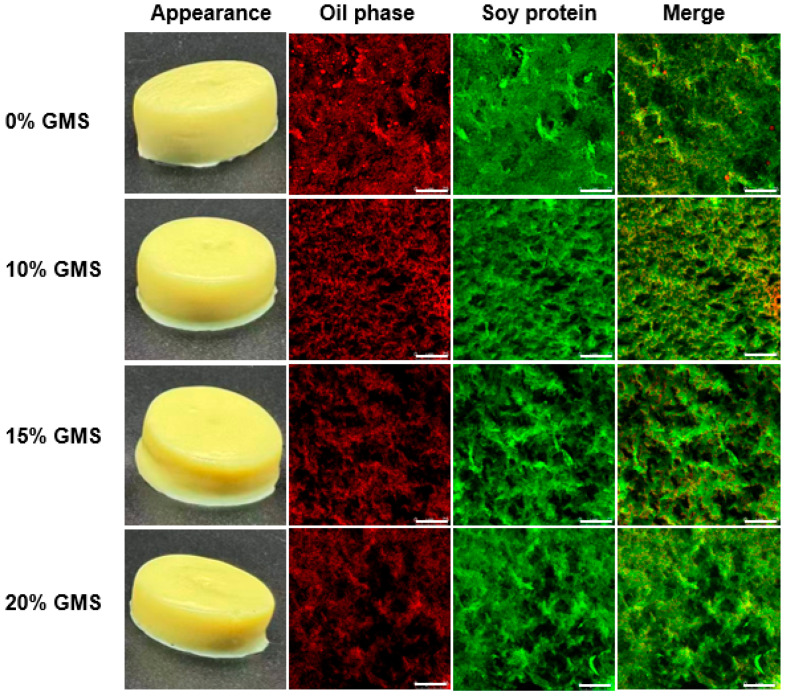
Appearance images and confocal laser scanning microscopy images of curcumin-loaded soy protein isolate-κ-carrageenan emulsion gels and bigels with various glycerol monostearate (GMS) concentrations. The red and green represent the oil phase and protein phase, respectively. The scale bar is 75 µm.

Figure 2 shows the visual appearance and microstructure of the curcumin-loaded SPI-κ-CG emulsion gels and bigels with various GMS concentrations. All formulations could form self-supporting gels with a yellow color. Generally, the emulsifier oil droplets were almost dispersed within the protein network in the absence and presence of GMS, suggesting that both oil droplets and proteins were co-localized during the gel formation. It should be noted that larger oil droplets could be observed in the SPI-κ-CG emulsion gel, indicating that the aggregation or coalescence of oil droplets occurred during the gelation. There was no significant oil coalescence in SPI-κ-CG bigels, which is consistent with the DLS results (Figure 1A).

### 2.3. Encapsulation Efficiency of Curcumin

Curcumin possesses very low hydrosolubility of 11 ng/mL, which can be overcome by encapsulating with protein particles and emulsions [4]. As shown in Table 1, the encapsulation efficiency of curcumin in SPI-κ-CG emulsions was around 93%. This result was consistent with goat whey protein-gum Arabic complex-stabilized emulsion (96%) [28], glutelin-stabilized emulsions (~94%) [29], and mussel protein-stabilized sunflower oil emulsions (~95%) [7]. GMS addition slightly increased the encapsulation efficiency of curcumin, as the oil gelation prevented the release and diffusion of curcumin from the oil to the aqueous phase [30]. The encapsulation efficiency of curcumin in emulsion gels and bigels was around 100% (Table 1), suggesting that curcumin was completely encapsulated in emulsified oil droplets or colloidal particles in the aqueous phase during the gel formation [31]. That is to say, curcumin molecules did not release from the gel system during the KCl and GDL double crosslinked gelation process, resulting from the low hydrosolubility of curcumin and complexation of curcumin with soy protein particles dispersed in the aqueous phase [32].

### 2.4. Textural Properties

The textural characteristics of SPI-κ-CG emulsion gels and bigels containing different GMS concentrations are shown in Table 2. Overall, the gel matrix structure, emulsified oil droplets, and their interactions primarily influenced the textural profile of emulsion-filled gels and bigels [33,34]. The gel hardness and chewiness of SPI-κ-CG emulsion gel were 1304 g and 392 g, respectively. The incorporation of 10% GMS initially led to a slight reduction in the mechanical strength of the bigels. This may be explained by the ability of GMS to partially displace proteins from the oil–water interface, thereby weakening the interactions between emulsified oil droplets and the gel matrix and resulting in lower overall hardness [11]. However, with further increase in GMS concentration, a moderate enhancement in gel strength was observed. This strengthening effect is likely attributable to the role of GMS as an oleogelator, which contributes to the formation of a more structured and robust oleogel network within the bigel system, thereby increasing its overall mechanical properties [16]. It should be noted that the double crosslinking SPI-κ-CG bigels still showed higher gel strength than SPI-agar-alginate double crosslinking emulsion gels (gel hardness ~360 g) [35] and whey protein-gellan gum double crosslinking emulsion gels (gel hardness ~123.5 g) [36] at similar protein and polysaccharide concentrations. Therefore, the KCl-GDL sequential double crosslinking is an effective approach for the improvement of both SPI-κ-CG emulsion gel and bigel strength, mainly attributed to the higher degree of crosslinking and intertwined double protein-polysaccharide networks. The addition of GMS slightly decreased the gel springiness and cohesiveness of SPI-κ-CG emulsion gels and bigels (Table 2). This was primarily due to oil gelation weakening interactions between emulsified oil droplets and the gel matrix, thereby reducing gel elasticity and cohesiveness [37].

### 2.5. Water Holding Capacity

**Figure 3:**
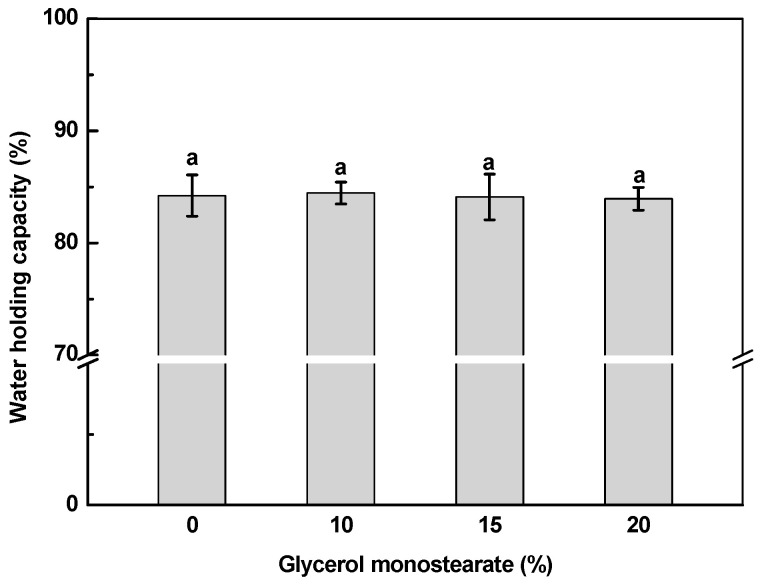
Water holding capacity of soy protein isolate-κ-carrageenan emulsion gels and bigels with various glycerol monostearate concentrations. Different letters indicate the statistically significant differences (*p* < 0.05).

Water holding capacity of SPI-κ-CG emulsion gels and bigels was around 84%, which was independent of the GMS concentration (Figure 3). The double crosslinked SPI-κ-CG emulsion gels and bigels showed higher water holding capacity than Ca^2+^-induced pea protein-κ-CG emulsion gels (~75%) [13]. The water holding capacity of gels is affected by the crosslinking methods due to the diverse gel microstructure [38,39]. The double crosslinking emulsion gels and bigels had denser networks and higher gel strength (Table 2) against water loss during centrifugation.

### 2.6. FTIR

**Figure 4:**
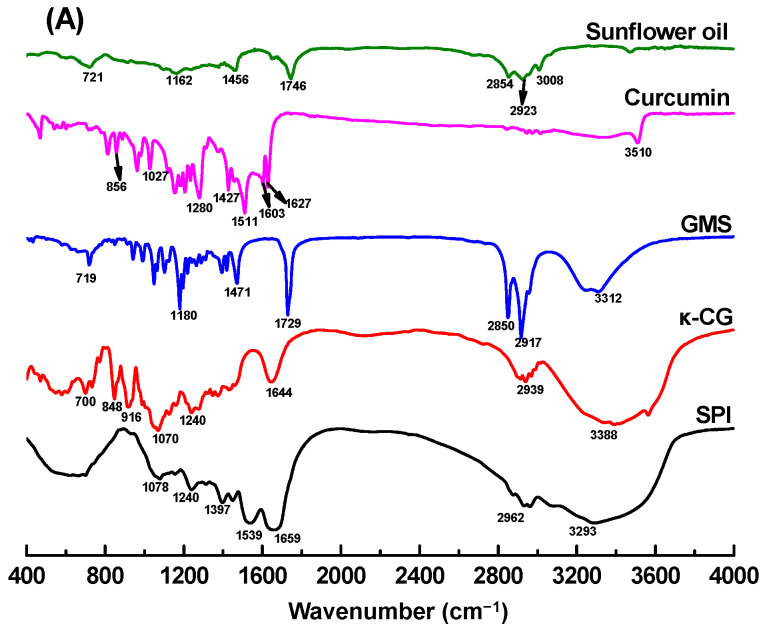

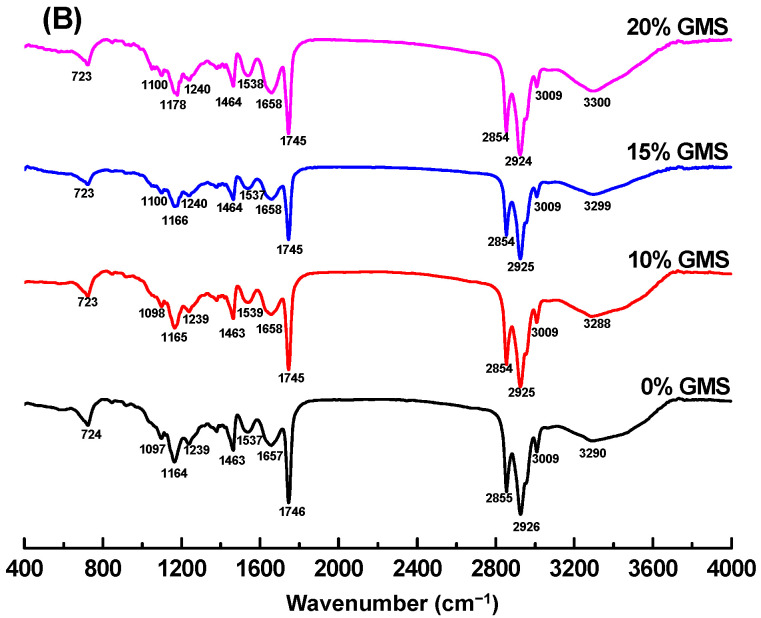
Fourier-transform infrared (FTIR) spectra of soy protein isolate (SPI), κ-carrageenan (κ-CG), curcumin, glycerol monostearate (GMS), and sunflower oil (**A**), as well as emulsion gels and bigels with various concentrations of GMS (**B**).

In Figure 4A, the broad absorption peaks at 3200~3600 cm^−1^ could be observed in curcumin (~3510 cm^−1^), GMS (~3312 cm^−1^), κ-CG (~3388 cm^−1^), and SPI (~3293 cm^−1^), indicating the presence of O-H stretching vibrations participating in hydrogen bonding. For the sunflower oil and GMS, peaks at 2800~3000 cm^−1^ corresponded to the stretching of C-H groups of triglycerides and monoglyceride [40]. SPI displayed two diagnostic peaks of 1659 cm^−1^ (amide I, C=O stretch) and 1539 cm^−1^ (amide II, N-H bend/C-N stretch). κ-CG exhibits characteristic peaks at 1644 cm^−1^ (carbonyl group stretching), 1240 cm^−1^ (ester sulfate group), 848 cm^−1^ (galactose-4-sulfate), 916 cm^−1^ (3,6-anhydro-D-galactose), and 1070 cm^−1^ (glycosidic bond) [41]. The FTIR spectrum of curcumin exhibited characteristic peaks in the regions 800~1627 cm^−1^, while no significant peaks were observed in the carbonyl region (1650~1800 cm^−1^), confirming that curcumin adopts the keto-enol tautomeric form [42].

FTIR analysis of curcumin-loaded emulsion gels and bigels (Figure 4B) revealed substantial attenuation of characteristic curcumin peaks, indicating successful compartmentalized encapsulation within emulsified oil droplets and protein particles during gel network formation [43]. The FTIR spectra of emulsion gels and bigels were similar to those of sunflower oil and GMS, attributing to the high proportion of the oil phase in the freeze-dried gel powders. Furthermore, the characteristic peaks of SPI and κ-CG were included in the spectrum of emulsion gels and bigels. No new peaks and drastic shifting were observed in the spectra of emulsion gels and bigels. It is therefore speculated that the SPI-κ-CG emulsion gels and bigels were mainly stabilized by the noncovalent bonds.

### 2.7. Intermolecular Force

**Figure 5:**
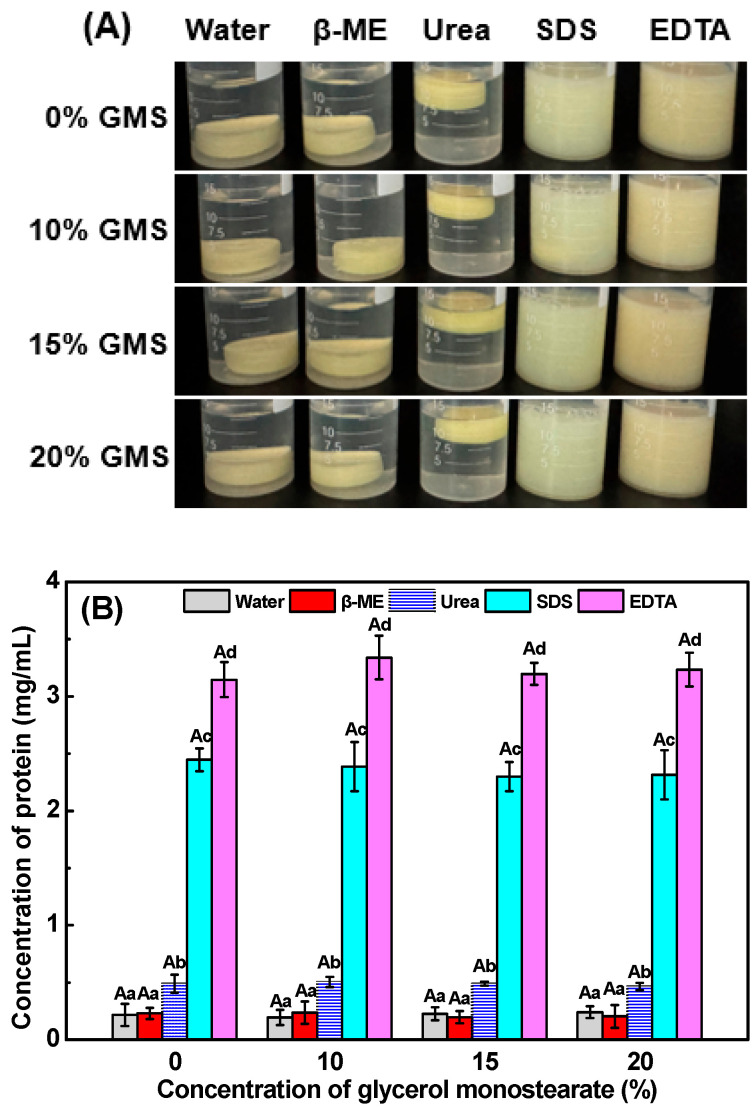
Appearance images (**A**) and protein solubility (**B**) of the emulsion gels and bigels with various concentrations of glycerol monostearate (GMS) in water, β-mercaptoethanol (β-ME), urea, sodium dodecyl sulfate (SDS), and ethylene diamine tetraacetic acid•2Na (EDTA). Different letters (uppercase letters for the samples with different GMS concentration in the same solvent, lowercase letters for the samples at the same GMS concentration in different solvents) indicate statistically significant differences (*p* < 0.05).

The significant gel disintegration (Figure 5A) and protein dissolution (Figure 5B) of all gel samples were observed in EDTA and SDS solutions, while no significant gel dissolution was observed in water, β-mercaptoethanol solution, and urea, indicating that electrostatic and hydrophobic interactions are the primary forces for maintaining SPI-κ-CG emulsion gels’ and bigels’ integrity. During the double crosslinking emulsion gel and bigel formation, the presence of K^+^ would induce the aggregation of the helices and κ-CG gel network formation through the hydrogen bonds and hydrophobic interactions by shielding the electrostatic repulsion of the κ-CG sulfate groups [44]. Meanwhile, GLD-induced pH decrease facilitates the electrostatic interactions between SPI and κ-CG to form the secondary networks. Therefore, the electrostatic and hydrophobic interactions play an important role in the SPI-κ-CG emulsion gel and bigel formation.

### 2.8. Chemical Stability of Curcumin in Emulsion Gels and Bigels

**Figure 6:**
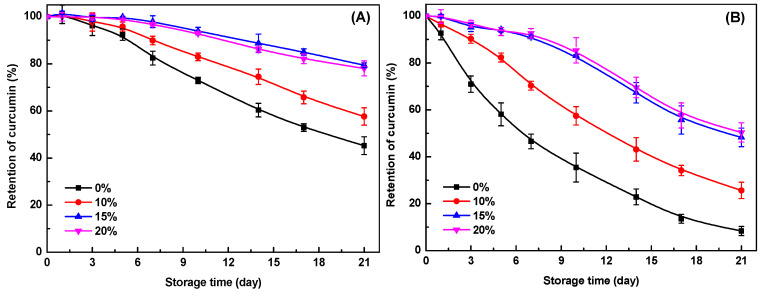
Retention of curcumin encapsulated within soy protein isolate-κ-carrageenan emulsion gels and bigels with various glycerol monostearate (GMS) concentrations during storage for 21 days at 25 °C (**A**) and 45 °C (**B**).

Curcumin is sensitive to environmental conditions (e.g., pH, light, and heat) and rapidly degraded in the aqueous phase [45]. As shown in Figure 6A,B, curcumin retention in SPI-κ-CG emulsion gels decreased progressively during storage at both 25 °C and 45 °C, with 45% and 8% remaining after 21 days, respectively. This high initial retention was due to curcumin encapsulation within the inner oil phase, which provided substantial protection from the external environment [46]. Furthermore, the pH decrease during GDL-induced gel formation enhanced curcumin’s physicochemical stability [45]. GMS incorporation further reduced curcumin degradation (Figure 6), demonstrating that the bigel structure offers enhanced protection. This effect intensified at higher GMS concentrations. At 20% GMS, curcumin retention reached ~78% at 25 °C and 50% at 45 °C after storage for 21 days (Figure 6A,B). These findings align with studies showing that entrapment within gel networks improves bioactive compounds’ stability [5]. In bigels, the concurrent gelation of both aqueous and lipid phases synergistically impedes ingredient degradation. The hydrogel matrix acts as a physical barrier against pro-oxidants and free radicals [47], while oleogel networks restrict molecular mobility and limit contact with environmental degradation factors [14]. Therefore, the encapsulation of curcumin in SPI-κ-CG bigels with high GMS concentration could effectively improve its chemical stability during storage.

### 2.9. In Vitro Digestion

#### 2.9.1. Free Fatty Acids Release

**Figure 7:**
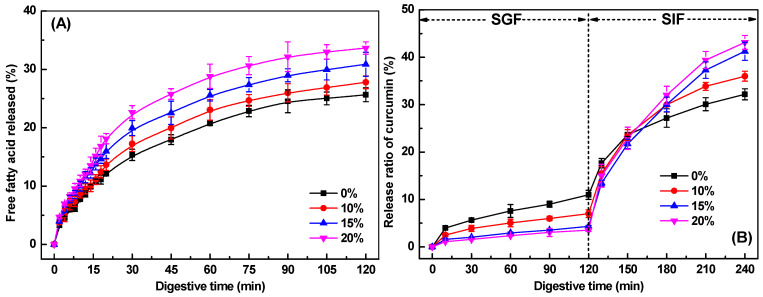
(**A**) The release of free fatty acids from soy protein isolate-κ-carrageenan emulsion gels and bigels with various glycerol monostearate concentrations during in vitro simulated intestinal digestion. (**B**) The release of curcumin from emulsion gels and bigels with various glycerol monostearate concentrations during simulated gastrointestinal digestion.

Figure 7A depicts free fatty acid (FFA) release profiles during intestinal digestion for SPI-κ-CG emulsion gels and bigels with varying GMS concentrations. All systems exhibited rapid initial FFA liberation followed by progressively slower release kinetics. Without GMS, about 15% FFAs were released within 30 min, increasing to just 26% by the end of intestinal digestion. Incorporation of 10% GMS slightly enhanced both release rate and extent, with values of 17% and 28% after 30 min and 120 min, respectively (Figure 7A). Further increase in GMS concentration resulted in a slight increase in lipid digestion rate and extent. As the GMS concentration reached 20%, there was a further increase in lipid digestion, with 23% and 34% of lipids being digested after 30 min and 120 min, respectively. These results suggested that the addition of GMS could improve the lipid digestion of bigels during in vitro intestinal digestion. The oelogel with three-dimensional networks has been expected to delay lipid digestion through inhibiting the liquid oil diffusion and the access of the lipase to oils, depending on the oil composition, oleogelator types, gelation mechanism, and the gel structure and strength [17,48,49]. However, it has been reported that GMS presented significantly higher digestibility than glyceryl tripalmitate, because pancreatic lipase could selectively hydrolyze GMS with fatty acids located at sn-1 or 3 positions [50]. Therefore, the increase in GMS concentration might contribute to the higher overall fatty acids release values.

#### 2.9.2. In Vitro Release Profile of Curcumin

Figure 7B displays curcumin release profiles from emulsion gels and bigels during simulated gastrointestinal digestion. Generally, a small fraction of curcumin was released (<11%) after SGF digestion, as curcumin was mainly present in the oil phase or oleogel phase. Minimal curcumin release (<4%) in bigels with 20% GMS occurred during the simulated gastric fluid (SGF), as most curcumin remained encapsulated within the oleogel phases. Bigels with higher GMS concentrations exhibited significantly slower release rates than emulsion gels, demonstrating enhanced gastric protection against curcumin degradation. This aligns with findings in whey protein-based [51], gelatin-based [16], and alginate-based emulsion gel and bigel systems [19].

Upon transition to simulated intestinal fluids, both emulsion gels and bigels showed rapid curcumin release (Figure 7B), primarily driven by protein and lipid hydrolysis. Notably, bigels with higher GMS concentrations ultimately released more curcumin than emulsion gels, which correlates with their higher FFA release (Figure 7A). The increased availability of FFAs promotes the formation of mixed micelles, thereby enhancing the solubilization and bioaccessibility of curcumin [50]. This improvement in bioaccessibility suggests a corresponding increase in curcumin bioavailability, demonstrating that modulating GMS content enables tailored control over nutraceutical release kinetics in bigel-based delivery systems.

The bigel system demonstrates excellent protection and controlled release of curcumin, making it suitable for enhancing the stability of hydrophobic bioactive compounds in functional food products, such as fortified beverages, yogurts, or health bars. Furthermore, the ability to suppress premature release in gastric conditions while enabling intestinal-specific liberation suggests potential for designing targeted nutrient delivery systems that improve absorption and efficacy. However, it should be noted that this study has certain limitations, including the absence of in vivo tests to evaluate the bioavailability and physiological efficacy of the released curcumin, as well as a lack of assessment regarding the sensory properties and stability of the bigels when incorporated into real food matrices. Future studies should therefore focus on validating the in vivo performance of curcumin-loaded SPI-κ-CG bigels and evaluating their sensory compatibility and stability in actual food applications.

## 3. Conclusions

This study successfully fabricated soy protein isolate-κ-carrageenan emulsion gels and bigels, demonstrating excellent curcumin encapsulation efficiency, protective capacity, and targeted delivery performance. Incorporating GMS to form hydrogel-in-oleogel bigel structures slightly decreased gel mechanical strength but significantly improved curcumin’s chemical stability during storage. Crucially, GMS-enriched bigels suppressed premature curcumin release in gastric conditions while enhancing intestinal-phase liberation. Comparative analysis revealed that the bigels, with their embedded oleogel phase, outperformed emulsion gels in encapsulating hydrophobic polyphenols like curcumin. These structures notably improved the compound’s resistance to degradation and enabled more efficient intestinal-specific release. The findings underscore the potential of SPI-κ-CG bigels as effective oral delivery systems for lipophilic bioactive compounds, offering promising applications in functional foods, nutraceuticals, and targeted nutrient delivery platforms.

## 4. Materials and Methods

### 4.1. Materials

Soy protein isolate (BR, 99%) was purchased from Shandong Xiya Reagent Co., Ltd. (Linyi, Shandong, China). Sunflower oil (Brand Duoli) was obtained from a local retailer (Wuxi, Jiangsu, China). κ-Carrageenan (molecular weight 300,000 g/mol), bile salts, porcine pancreatin (4 × USP specifications), Nile Red, Nile Blue, β-mercaptoethanol, and porcine pepsin (≥500 U/mg) were purchased from Sigma-Aldrich Co., Ltd. (St. Louis, MO, USA). Glycerol monostearate, sodium dodecyl sulfate (SDS), HCl, ethylene diamine tetraacetic acid (EDTA), and NaOH were of analytical grade and obtained from SinoPharm CNCM Ltd. (Shanghai, China). All samples were formulated with deionized water purified using a Milli-Q purification system (Millipore, Billerica, MA, USA).

### 4.2. Emulsion Preparation

A 5 wt% soy protein isolate (SPI) solution was subjected to thermal denaturation at 90 °C for 30 min, followed by controlled cooling to room temperature. Separately, a 0.5 wt% κ-carrageenan (κ-CG) stock solution was formulated by dissolving κ-carrageenan powder in pure water at 55 °C with continuous stirring for 2 h. Glycerol monostearate (0, 10, 15, or 20 wt% based on oil weight) was incorporated into sunflower oil at 75 °C under agitation. Curcumin (0.5 mg/g oil phase) was dispersed in the lipid phase. Primary emulsions were formulated via homogenization of a 90 wt% SPI dispersion with a 10 wt% oil phase using a high-speed homogenizer (Ultra-Turrax T25, IKA, Staufen, Germany) at 12,000 rpm for 2 min, followed by high-pressure homogenization (AH-2010, ATS Engineering, Brampton, ON, Canada) under 50 MPa pressure and at 50 °C for 2 min. The resulting emulsion was combined with an equal-volume κ-CG solution under continuous stirring (55 °C, 30 min). Final compositions contained 2.5 wt% SPI, 5 wt% oil phase, and 0.25 wt% κ-CG.

### 4.3. Emulsion Size Distribution and ζ-Potential Measurement

The particle size distribution and ζ-potential of emulsion samples were measured via dynamic light scattering (DLS) using a Brookhaven Instruments Co., Ltd. (New York, NY, USA) analyzer at 25 °C after suitable dilution. For particle size analysis, intensity distributions were derived from measurements conducted at a 90° scattering angle. ζ-Potential values were computed using the Smoluchowski theory.

### 4.4. Fabrication of Emulsion Gels and Bigels

The freshly prepared emulsion was mixed with 1 M KCl to achieve a final gel concentration of 10 mM KCl. After overnight storage at 4 °C for gelation, KCl-induced emulsion gels/bigels were immersed in twice the volume of 1 wt% GDL solution and incubated at 4 °C. This yielded dual-crosslinked gels/bigels through successive KCl and GDL crosslinking mechanisms.

### 4.5. Encapsulation Efficiency of Curcumin in the Emulsions and Gels

Curcumin content in emulsified oil droplets, emulsion gels, and bigels was quantified following established protocols [52]. Emulsions were centrifuged at 20,000× *g* for 30 min. The supernatant (0.5 mL) and GDL solution (0.5 mL) used for the gel preparation were mixed with 9.5 mL ethanol to extract curcumin, followed by centrifugation at 10,000× *g* for 10 min. Curcumin concentration in ethanol extracts was determined at 425 nm using a Shimadzu UV-Vis spectrophotometer (Tokyo, Japan) against a standard curve (*y* = 137.73*x* − 0.0017, *r*^2^ = 0.9996). Encapsulation efficiency was calculated using Equation (1):

(1) $$\mathrm{Encapsulation}\;\mathrm{efficiency}\;(\%)\;=\;\left(1-\frac{C_{a}}{C_{t}}\right)\;\times \;100\%$$

where *C_t_* represents total curcumin content in whole emulsion, emulsion gels, and bigels, *C_a_* represents curcumin content in the supernatant and GDL solution during gelation.

### 4.6. Water Holding Capacity (WHC)

WHC was determined using an established method [53]. Approximately 3 g gel samples were centrifuged (8000× *g*, 20 min) in 50 mL tubes. Excess water was removed with filter paper, and WHC was calculated using Equation (2):

(2) $$\mathrm{WHC}\;(\%)\;=\;\left(1-\frac{W_{t}\;-{\;W}_{1}}{W_{t}}\right)\;\times \;100\%$$

where *W_t_* represents the initial gel mass (g), *W*_1_ represents gel mass after centrifugation and water removal (g).

### 4.7. Texture Profile Analysis (TPA)

TPA was conducted using a TA.XT Plus texture analyzer (Stable Micro Systems, Surrey, UK) equipped with a P-36R cylindrical probe [35]. Cylindrical samples (2 cm diameter × 1.5 cm height) were compressed twice to 50% strain at 1.0 mm/s with a 3 g trigger force. Textural parameters were derived from the resultant force-time curves using Exponent software (Version 8).

### 4.8. Microstructure Characterization

The microstructure of samples was observed using a TCS SP8 confocal laser scanning microscope (Leica Microsystems GmbH, Heidelberg, Germany). Nile Red (2 mg/mL, ethanol, dyes in oil phase) and Nile Blue (2 mg/mL, distilled water, dyes in protein phase) were added to the emulsion at ratios of 1:50 and 1:100 (*v*/*v*, dye: emulsion), respectively, before the gel preparation. The dyeing gel samples were placed on a slide and covered with a coverslip. At excitation spectra of 633 nm for Nile Blue and 552 nm for Nile Red, images of the regions represented by each sample were taken with 40× (objective lens) magnification using an He-Ne laser.

### 4.9. Fourier Infrared Spectroscopy (FTIR)

Freeze-dried gel samples were ground with KBr in a 1:100 (*w*/*w*) mass ratio and homogenized to form a uniform powder. FTIR spectral data were collected using a Nicolet iS10 Fourier-transform infrared (FTIR) spectrometer (Thermo Fisher Scientific, Waltham, MA, USA). Spectra were recorded from 4000~400 cm^−1^ at 4 cm^−1^ resolution (32 scans co-added), with background subtraction using pure KBr.

### 4.10. Intermolecular Force Analysis

Intermolecular forces stabilizing emulsion gels and bigels were characterized using an established solvent-disruption protocol [54]. Gel samples (2.5 g) were immersed in 10 mL of target solvents and continuously dissolved at 25 °C for 3 h. Post dissolution, residual gels were filtered to obtain solubilized fractions. Four solvent systems were employed: pure water, Tris buffer containing 4 mmol/L EDTA·2Na (0.086 mol/L Tris, 0.09 mol/L glycine, pH 8.0; disrupting electrostatic interactions), 2% (*w*/*v*) SDS (disrupting hydrophobic interactions), 8 mol/L urea (disrupting hydrogen bonding), and 1% (*v*/*v*) β-mercaptoethanol (disrupting disulfide bonds). Soy protein concentrations in each solubilized fraction were quantified via the Kjeldahl method (N × 6.25 conversion factor).

### 4.11. Stability of Curcumin in Emulsion Gels and Bigels

All samples underwent accelerated stability testing at 25 °C and 45 °C for 21 days. The curcumin content in gel samples was determined using previously published protocols [52,55]. Briefly, 0.5 g gel samples were homogenized with 9.5 mL ethanol to extract curcumin, then centrifuged at 20,000× *g* for 30 min. The supernatant was analyzed at 425 nm using UV-Vis spectrophotometry. The stability of curcumin in emulsion gels and bigels was assessed by its retention throughout storage.

### 4.12. In Vitro Digestion of Emulsion Gels and Bigels

#### 4.12.1. Simulated Gastrointestinal Tract Digestion

Gel samples underwent in vitro digestion following a modified INFOGEST 2.0 protocol [56]. Briefly, 10 g of gel samples were minced and dispersed in 10 mL of PBS (pH 7.0) for 3 min. Subsequently, the resulting mixture was incubated with simulated gastric fluid (SGF) containing 2000 U/mL pepsin at pH 3.0 over a 2 h period. Following SGF incubation, the digesta were combined with 40 mL of simulated intestinal fluid (SIF) containing 100 U/mL pancreatin and 10 mM bile salts, and mixed for 2 h. The pH of the digestive fluid was maintained at 7.0 by incremental addition of 0.1 M NaOH throughout the entire intestinal digestion phase. Quantification of free fatty acid (FFA) release was performed using Equation (3) [57,58]. All digestions occurred at 37 °C with constant agitation (100 rpm).

(3) $$\mathrm{FFA}\;(\%)=\frac{V_{\mathrm{NaOH}}\;\times \;C_{\mathrm{NaOH}}\;\times \;M_{\mathrm{Lipid}}}{{2\;\times \;W}_{\mathrm{Lipid}}}\;\times \;100\%$$

where *V_NaOH_* represents volume of NaOH titrant consumed (mL), *C_NaOH_* represents molarity of NaOH solution (0.1 M), *M_Lipid_* represents average molecular weight of sunflower oil triglycerides (880 g/mol), and *W_Lipid_* represents mass of lipids in digested samples (g). The stoichiometric relationship arises from lipase-catalyzed hydrolysis, where each triacylglycerol molecule releases two free fatty acids.

#### 4.12.2. Release Profile of Curcumin During the Digestion

To evaluate curcumin release kinetics from emulsion gels and bigels, digestive aliquots were collected every 30 min during gastrointestinal simulation. Each sample was immediately quenched in ice-water to halt enzymatic activity. Curcumin content in quenched aliquots was quantified per Section 2.5 methodology. Cumulative release was calculated using Equation (4):

(4) $$\mathrm{Release}\;\mathrm{ratio}\;\mathrm{of}\;\mathrm{curcumin}\;(\%)\;=\;\frac{C_{t}}{C_{i}}\;\times \;100\%$$

where *C_i_* represents the initial content of curcumin in emulsion gels or bigels, *C_t_* represents the content of curcumin at time *t* during digestion.

### 4.13. Statistical Analysis

Each experiment was conducted in at least triplicate replicates. The results are expressed as mean ± standard deviation (SD). Statistical analysis was performed using one-way analysis of variance (ANOVA) combined with Dunnett’s post hoc test, executed via SPSS 20.0 software (IBM Corporation, New York, NY, USA). A *p*-value less than 0.05 was considered statistically significant.

## Figures and Tables

**Table 1:** Encapsulation efficiency of curcumin within soy protein isolate-κ-carrageenan emulsions, emulsion gels, and bigels with various glycerol monostearate (GMS) concentrations.

GMS (%)	Encapsulation Efficiency (%)	
	Oil Droplet of Emulsion	Gel Sample
0	93.2 ± 0.9 ^a^	98.2 ± 0.7 ^a^
10	95.5 ± 1.1 ^b^	99.1 ± 0.9 ^a^
15	96.4 ± 0.8 ^b^	98.8 ± 0.4 ^a^
20	97.1 ± 1.0 ^b^	99.3 ± 0.3 ^a^

Different letters in the same column indicate statistically significant differences (*p* < 0.05).

**Table 2:** Textural characteristics of soy protein isolate-κ-carrageenan emulsion gels and bigels with various glycerol monostearate (GMS) concentrations.

GMS (%)	Hardness (g)	Springiness	Cohesiveness	Chewiness (g)
0	1304.6 ± 24.7 ^a^	0.594 ± 0.025 ^a^	0.506 ± 0.027 ^a^	392.9 ± 32.3 ^a^
10	940.5 ± 38.9 ^b^	0.592 ± 0.014 ^a^	0.465 ± 0.001 ^ab^	259.1 ± 21.1 ^b^
15	1117.8 ± 30.3 ^c^	0.573 ± 0.022 ^ab^	0.429 ± 0.021 ^bc^	274.7 ± 6.7 ^b^
20	1285.8 ± 15.8 ^c^	0.544 ± 0.003 ^b^	0.414 ± 0.037 ^c^	289.6 ± 30.3 ^b^

Different letters in the same column indicate statistically significant differences (*p* < 0.05).

## Data Availability

The original contributions presented in this study are included in the article. Further inquiries can be directed to the corresponding author.
